# Exploring Finger Digit Ratios (2D:4D) in Surgeons, Professional Rugby Players, and Political Journalists to Form a Directional Hypothesis: Could Finger Length Predict Attention and Focus?

**DOI:** 10.3389/fnbeh.2022.873129

**Published:** 2022-04-27

**Authors:** Benjamin G. Serpell, Christian J. Cook

**Affiliations:** ^1^ACT Brumbies, Canberra, ACT, Australia; ^2^Geelong Cats Football Club, Geelong, VIC, Australia; ^3^School of Science and Technology, University of New England, Armidale, NSW, Australia; ^4^Hamlyn Centre, Imperial College, London, United Kingdom

**Keywords:** digit ratio, hormones, testosterone, 2D:4D, stress, performance, achievement

## Abstract

In this short report we explore the predictive nature of finger digit ratio (i.e., second/index finger length divided by fourth/ring finger length; 2D:4D) and achievement. This research, with niche and specialized populations, was intended to support and grow on knowledge obtained from other large population 2D:4D studies and help form a directional hypothesis for future work exploring finger digit ratio and “success.” Twenty-nine professional rugby players aged 25.1 ± 4.2 years, height 185.2 ± 6.3 cm and weight 101.9 ± 11.8 kg; *n* = 16 orthopedic surgeons aged 55.3 ± 9.3 years with height 183.8 ± 10.2 cm and weight 90.8 ± 14.0 kg; and *n* = 18 political journalists with age, height and weight of 38.8 ± 7.3 years, 182.8 ± 7.8 cm, and 84.4 ± 11.4 kg, respectively, were recruited. Three experiments were conducted where we (1) explored relationships for 2D:4D with testosterone and cortisol responsiveness to low stress exercise, (2) explored relationships for 2D:4D with pupil constriction and pupil constriction latency (pupillometry measures related to testosterone and cortisol responsiveness and to attentiveness), and (3) compared 2D:4D between rugby players, surgeons, and journalists. Our results revealed 2D:4D was not predictive of testosterone and cortisol responsiveness to low-level exercise stress. However, relationships exist for 2D:4D and pupillometry measures (*p* < 0.05). Journalists right minus left 2D:4D difference was significantly different to rugby players’ and surgeons (*p* < 0.05). We argue 2D:4D is likely predictive of testosterone sensitivity and associated ability to focus attention; a skill important to high achievement in various contexts.

## Introduction

Research in physical and social sciences has looked to physical and biological markers to predict or explain human behavior and performance (Alonso et al., 2018). One such observation is right hand second finger digit length relative to right hand fourth finger digit length, or second-to-fourth digit ratio (2D:4D; Manning et al., 2014; Alonso et al., 2018). The digit ratio is thought to be established during early prenatal development. Specifically, prenatal testosterone exposure is known to influence sexual dimorphism *in utero* (including 2D:4D) as early as the end of the first trimester (Manning et al., 2014). Unsurprisingly, therefore, females typically have a higher 2D:4D ratio than males (Manning et al., 2014; Alonso et al., 2018). Although the age at which right 2D:4D “stabilizes” is unknown, once it does it is constant throughout life (Jeevanandam and Muthu, 2016) with change generally only through structural deformation such as when trauma to the hand occurs. Additionally, prenatal testosterone exposure has organizing effects on the developing brain, and it potentially influences testosterone responsiveness later in life (Coates et al., 2009; Coco et al., 2011; Alonso et al., 2018). Consequently, it is hypothesized that right 2D:4D can be predictive of testosterone responsiveness and, by extension, certain human behavior and performance. This seems reasonable given digit ratio may be linked to androgen receptor gene structure (Manning et al., 2003; Zhang et al., 2020); and testosterone response in some high stress challenges appears predicted by a low 2D:4D (Kilduff et al., 2013a,b; though it may not be in low-stress scenarios (Kowal et al., 2020).

Testosterone is a hormone from the androgen group with cross regulation by glucocorticoid hormones, such as cortisol (Cook and Crewther, 2012). Cortisol is generally considered a stress hormone which can be arousing, however, it can also have an opposing functional outcome to testosterone; leading to increased aggressive behaviors (Papacosta et al., 2016) and in prolonged doses may also increase fatigue and anxiety (Mehta and Josephs, 2010; Cook and Crewther, 2012; Crewther et al., 2017). High levels of cortisol may also affect ability to receive and process information as well as execute motor tasks (Serpell et al., 2020b; Serpell and Cook, 2021). Due to the highly interconnected nature of these hormones they often can be examined relative to each other using testosterone to cortisol ratio (i.e., testosterone divided by cortisol; T:C; Cook and Crewther, 2012), and it has been argued that a response which promotes an increase in testosterone of greater magnitude than cortisol (i.e., increased T:C) is favorable in many contexts (Strahorn et al., 2017; Serpell et al., 2018).

Functional outcomes which may be observed with increases in testosterone and T:C can include enhanced physical work output (Smith et al., 2013; Crewther et al., 2015b; Russell et al., 2016), improved chronic training adaptation (Crewther et al., 2011), emotion change (Liening and Josephs, 2010; Cook and Crewther, 2012, 2019; Crewther and Cook, 2018), enhanced cognitive ability and memory (Celec et al., 2015; Serpell et al., 2020b), improved motor function (Serpell et al., 2020b), and enhanced competitive performance by means of increased assertiveness, and increased cohesion within a team combined with collective aggression toward opposition (Cook and Crewther, 2014, 2019; Gaviglio et al., 2014; Russell et al., 2016; Mehta et al., 2017; Crewther and Cook, 2018). The mechanisms by which these outcomes occur include activation of signaling pathways which promote mobilization of energy stores (Sato et al., 2008; Mitsuhashi et al., 2016), modulation of neuromotor units (Bonifazi et al., 2004), the accumulation of protein for muscle “growth” via mTor (Basualto-Alarcon et al., 2013), and increased motivation (Smith et al., 2013; Cook et al., 2021). Therefore, given the role testosterone can play when it comes to human behavior and performance, the mechanisms by which it works, and that prenatal testosterone exposure may influence testosterone responsiveness later in life, it is unsurprising to see that right 2D:4D has been shown to be predictive of social and competitive behaviors (Kilduff et al., 2013a,b; Crewther et al., 2015a; Nepomuceno et al., 2016), sporting performance (including in international Rugby union, distance running, skiing, and sumo wrestling; Manning, 2002, Manning et al., 2007, 2014; Tamiya et al., 2012; Kilduff et al., 2013a; Lombardo and Otieno, 2020), musical ability (Sluming and Manning, 2000), risk taking behaviors (Coates et al., 2009; Massimino et al., 2019), and academic performance (Coco et al., 2011; Tektas et al., 2019).

Although being linked to performance on many specific tasks primarily physical, it is important to note that recent research has also shown a link between 2D:4D and “good luck” but that this link was due to chance (Smoliga et al., 2021). Thus, we cannot simply associate 2D:4D as a biomarker for “nearly everything” (Smoliga et al., 2021), however, given that which was discussed in the previous paragraph it is speculatively possible that right 2D:4D can be associated with relative levels of achievement, independent of domain, context or profession where motivational and assertive behaviors are involved (Millet and Dewitte, 2008). This is because of the likely association between 2D:4D and testosterone responsiveness given the link between right 2D:4D, free testosterone and potentially androgen receptor gene structure (Manning et al., 2003). It has been hypothesized that people with low right 2D:4D are better responsive to feedback (Millet and Dewitte, 2008), and it is also pertinent to note that high levels of testosterone relative to cortisol may increase an individuals’ focus/attentiveness (Serpell et al., 2019, 2020b; Lightman et al., 2020; Serpell and Cook, 2021). Therefore, a low right 2D:4D may be common to high achievers regardless of whether they are elite athletes or specialist doctors. That is, based on right 2D:4D, and assuming effects of androgenic hormone responsiveness, it is possible that an orthopedic surgeon has similar patterns of response to a fly-half in international Rugby union.

The purpose of this work was to explore further the relationship between digit ratios and high achievement. Three small experiments were performed; firstly, we explored whether a relationship for testosterone and cortisol production with digit ratios exist in a group of professional rugby players using a relatively low-level stress to which they were highly familiar. Secondly, because testosterone and cortisol responsiveness may be related to proxy measures of receiving and processing information (e.g., cognition via pupil reflex; Serpell et al., 2019, 2020b), we explored whether digit ratios were related to pupillometry in a high performing population (also professional rugby players). Research in healthcare, military and with people with traumatic brain injury has argued increased pupil dilation (or a greater pupil constriction) is associated with increased attention; and slower onset of pupil constriction (i.e., increased pupil constriction latency) suggests delayed processing of information (Capó-Aponte et al., 2013; Thiagarajan and Ciuffreda, 2015; Lynch, 2018; Bicalho et al., 2019; Cabrera-Mino et al., 2019; McGrath et al., 2019). Finally, we compared digit ratios between varying populations (professional rugby players versus orthopedic surgeons versus political journalists). Most work exploring digit ratios and human biomarkers and functional performance is limited to right hand digit ratio only, this may be because right 2D:4D correlates better to target traits than does left 2D:4D (Manning et al., 2014). However, there is some evidence that right minus left digit ratio can predict free testosterone concentration (Kilduff et al., 2013a), and that a negative figure in this measurement can predict reactive aggression and is associated with traits more typical of males (Coyne et al., 2007). Therefore, we explored both left and right digit ratios. We hypothesized that digit ratios would not be related to testosterone reactivity in a mild familiar stress (study 1), but that we would see a relationship between right digit ratio and pupil reflex – a physical behavior which may reflect ongoing androgen responsiveness/sensitivity (study 2). Finally, we expected to see some differences in digit ratios between people of different professions (professional rugby players, orthopedic surgeons, and experienced print media political journalists) due to difference in demands of behaviors and skills for success (study 3). It was anticipated that outcomes could provide a directional hypothesis for future work exploring digit ratio and relative success and contribute to the current 2D:4D knowledge base obtained from other large population 2D:4D studies.

## Methods

### Study Overview

All three studies were observational in nature. Studies one and two were correlational, with study one examining an association for salivary testosterone (sal-T), salivary cortisol (sal-C) and T:C responsiveness to physical exercise with right 2D:4D, left 2D:4D, and right minus left 2D:4D. Study two examined a correlation between left pupil dilation and pupil constriction latency with direct and consensual stimulus, with right 2D:4D, left 2D:4D, and right minus left 2D:4D. Finally, study three was a cross-section cohort comparison study; we compared right 2D:4D only between consultant orthopedic surgeons, professional rugby players, and print media political journalists; all subjects were regarded, by both position and peers, as successful in their careers. We also deemed this reasonable given each of those professions are highly specialized; to become a qualified orthopedic surgeon requires outstanding academic merit and approximately 15 years of practical training. Many people participate in rugby, however, very few are deemed skilled enough to be recruited and paid by professional rugby clubs; in fact, those recruited to this study would be ranking in the top 100 players in Australia. Finally, many people become qualified in journalism, however, the opportunity to be employed in the capacity that journalists who participated in this study are employed is considerably limited. Ethical approval to conduct this research was granted by the (blinded for review) human research ethics committee.

### Participants

Thirteen professional rugby players aged 26.4 ± 2.6 years (mean ± SD) with height and weight 186.5 ± 6.6 cm and 105.9 ± 11.4 kg, respectively, were recruited for study one. For study two, 20 professional rugby players aged 24.8 ± 4.8 years and 185.1 ± 6.6 cm and 101.4 ± 13.5 kg were recruited. Finally, for study three, three cohorts were recruited – *n* = 29 male professional rugby players aged 25.1 ± 4.2 years with height and weight 185.2 ± 6.3 cm and 101.9 ± 11.8 cm, respectively; *n* = 16 male consultant orthopedic surgeons aged 55.3 ± 9.3 years with height 183.8 ± 10.2 cm and weight 90.8 ± 14.0 kg; and *n* = 18 male print media political journalists with age, height, and weight of 38.8 ± 7.3 years, 182.8 ± 7.8 cm, and 84.4 ± 11.4 kg, respectively. Rugby players were recruited from a single professional rugby club which competes in the Super rugby competition, orthopedic surgeons were consultants who operate in a capital city in Australia, and political journalists were recruited from several major news-paper agencies in Australia from offices in the Australian Parliament House press gallery. Some participants in the rugby population participated in all three studies, some did not. These populations were selected because of their diversity from each other, and participants were conveniently sampled from each population. No participants report a history of injury to the hands; and no participants in study two had a recorded history of concussion.

### Procedures

To measure digit ratios participants were asked to place the ventral surface of their left and right hands on the glass of a photocopier without pressing down to not distort the fingertips. Using a set of vernier calipers (Mentone Educational, VIC, Australia) second and fourth finger length on left and right hands were measured from photocopies of the hands from the mid-point of most proximal finger crease (the crease where the finger meets the palm) to the most distal point of the finger. Additional measures are described below.

#### Study 1

Following a day free from training, participants completed a whole-body gym-based speed-power-strength (SPS) session. The SPS training session was 60-min in duration and consisted of a standardized warm up followed by reactive strength, low-load speed strength, high-load speed strength, and maximum strength exercises. The session has been described in more detail elsewhere (Serpell et al., 2018). While this session would be stressful to a recreational athlete it was hypothesized as only of potentially low stress to these professional athletes. Passive drool saliva samples were collected from each participant prior to commencement of the SPS training session and again 15 min after the session. Participants were instructed to not eat or drink anything in the 30 min prior to providing saliva samples and were also instructed to rest between completion of the training session and providing the post-training saliva sample. Participants were permitted to massage their gums to increase saliva flow.

Saliva samples were collected from participants in sterilized cryovials and stored in a –20^°^C freezer until analysis. They were then assayed for cortisol and testosterone using commercial enzyme immunoassay kits (cortisol catalog number 1-3002; testosterone catalog number 1-2402; Salimetrics LLC, State College, PA, United States). Testosterone assay sensitivity was 11.01 pmol/L with intra-assay and inter-assay variability of <4.4 and <4.9%, respectively. Cortisol assay sensitivity was 0.01 nmol/L with intra-assay and inter-assay variability of <5.6 and <6.2%, respectively. Samples were analyzed in the same assay to eliminate inter assay variance.

#### Study 2

Left eye pupil reflex was measured on the visual-vestibular test (VVT); a test which stimulates, records and analyses eye movements and the pupil reflex. For the VVT participants wore a commercially available Virtual Reality (VR) headset (FOVE, Inc, Torrance, CA, United States) that was used to deliver the stimulus and capture responses. The headset was equipped with a 2,560 × 1,440 pixel display with a video frame rate of 70 Hz and a field of view up to 100 degrees; coupled with dual IR cameras with video frame rate of 120 Hz. Participants were seated with their visual fields fully occluded by the VR headset and instructed to keep their head still and to look straight ahead. A small red cross was displayed in the center of the participant’s visual field as a focal point to assist the participant with maintaining a steady gaze during the stimulation cycles. A stimulation cycle consisted of a green circle displayed for a 1 s duration followed by 3 s of darkness. The green circle appeared in a different location of the display to each eye separately across the stimulation period. The changing diameter of the participant’s left eye pupil in response to the stimulus in both eyes was recorded for further analysis. The left eye pupil response with the right eye stimulus was considered “consensual” stimulus, the left eye pupil response with left eye stimulus was considered “direct” stimulus. Each participant was subjected to eight central target stimulation events and the pupil diameter data for the left eye in response to the central target displayed to both the direct and consensual stimulus was analyzed offline. The parameters selected for analysis were based on those determined by Capó-Aponte et al. (2013) as they are considered the most sensitive measures of cognitive deficit time delay between when the stimulus occurs and the subject’s pupil begins to contract in response, and magnitude of the pupil contraction. Pupillometry measurement error has been reported to be 0.30 mm (Lynch, 2018; McGrath et al., 2019).

#### Study 3

No measures other than finger length were taken. Simply, a comparison of digit ratios between populations was made.

### Statistical Analyses

#### Study 1

To establish whether data satisfied the assumptions for parametric statistical analysis a Shapiro–Wilk test was first used to determine if data was normally distributed. A Levene’s test for homogeneity of variance was then performed on data which was normally distributed. Only T:C change and right 2D:4D met the assumptions for parametric statistical analysis, therefore a series of Mann–Whitney *U* tests were performed to determine any significant differences between percentage change of sal-T, sal-C, and T:C. A Pearson correlation was then used to determine if a relationship for T:C change and right 2D:4D. Spearman’s correlations were used to establish if relationships exist amongst all other hormone and digit ratio variables.

#### Study 2

Similarly, to study one, for statistical analysis data was first tested to establish if the assumptions for parametric statistical analysis were met, starting with a Shapiro–Wilk’s test. Normality was confirmed for all variables except left eye constriction latency from consensual stimulus, and left eye constriction latency average from direct and consensual stimulus. Levene’s test for homogeneity of variance showed equality of variance for all pupillometry data, however, equality of variance was not seen for any digit ratio data. Therefore, a one-way ANOVA with Bonferroni *post hoc* was used to determine differences in means for left eye constriction from direct, consensual, and average of direct and consensual stimulus. A series of Mann–Whitney *U* tests were used to determine differences for constriction latency. Effect size was reported where significant differences exist. Finally, a series of spearman’s correlations were used to establish if relationship exist amongst all pupillometry and digit ratios.

#### Study 3

As in study one and study two, a Shapiro–Wilk’s test was used to confirm normal distribution for right 2D:4D, left 2D:4D and right 2D:4D minus left 2D:4D for the rugby players, the orthopedic surgeons, and the journalists. A Levene’s test for homogeneity of variance was also performed followed by a Mauchly’s test for sphericity. All data satisfied the assumptions for parametric statistical analysis and a one-way ANOVA with Bonferroni *post hoc* was performed to compare means for right 2D:4D, left 2D:4D and right 2D:4D minus left 2D:4D for rugby players versus orthopedic surgeons versus political journalists, and establish where differences exist.

All statistical analyses α = 0.05, and all were performed Statistical Package for the Social Sciences software version 26.0 (IBM, New York, NY, United States).

## Results

### Study 1

**FIGURE 1:**
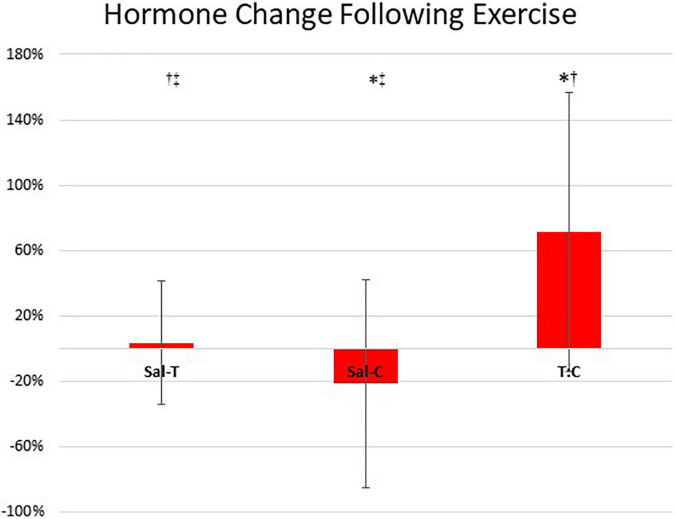
Study 1 hormone responsiveness to gym-based speed, power, and strength session as a proportion of pre-training values (mean ± standard deviation). Sal-T, salivary testosterone (pmol/L); sal-C, salivary cortisol (nmol/L); and T:C, testosterone to cortisol ratio [i.e., sal-T (pmol/L) divided by sal-C (nmol/l)]. *Significantly different to sal-T (*p* < 0.05). ^†^Significantly different to sal-C (*p* < 0.05). ^‡^Significantly difference to T:C (*p* < 0.05). No statistically significant relationships were observed for hormone responsiveness and digit ratios.

As was anticipated, in response to exercise change in sal-T was greater than change in sal-C (*U* = 34, *p* = 0.09), change in sal-C was less than change in T:C (*U* = 18, *p* = 0.00), and change in sal-T was lower than change in T:C (*U* = 36, *p* = 0.01). However, hormone changes were of small magnitude, particularly cortisol. No significant relationship was observed for right 2D:4D with change in sal-T [*r*_*s*_(11) = 0.01, *p* = 0.48], change in sal-C [*r*_*s*_(11) = –0.10, *p* = 0.37], and change in T:C [*r* = –0.03, *n* = 13, *p* = 0.46]; for left 2D:4D with change in sal-T [*r*_*s*_(11) = –0.18, *p* = 0.28], change in sal-C [*r*_*s*_(11) = –0.06, *p* = 0.42], and change in T:C [*r*_*s*_(11) = 0.06, *p* = 0.42]; or right 2D:4D minus left 2D:4D with change in sal-T [*r*_*s*_(11) = 0.14, *p* = 0.32], change in sal-C [*r*_*s*_(11) = –0.20, *p* = 0.28], and change in T:C [*r*_*s*_(11) = 0.16, *p* = 0.30] (Figure 1).

### Study 2

For pupil constriction there was no significant difference between direct stimulus, consensual stimulus, or average of direct and consensual stimulus [*F*(2,57) = 0.375, *p* = 0.69]. There was also no significant difference in left eye constriction latency between direct and consensual stimulus (*U* = 181.5, *p* = 0.62), between direct stimulus and average of direct and consensual stimulus (*U* = 189.5, *p* = 0.78), or between consensual stimulus and average of direct and consensual stimulus (*U* = 188, *p* = 0.76; Table 1). A significant moderate to strong correlation was seen for right 2D:4D with left eye pupil constriction difference between direct and consensual stimulus. That is, a smaller difference in left eye pupil constriction between direct and consensual stimulus was positively associated with right 2D:4D. Further, a significant moderate to strong inverse relationships were seen for right 2D:4D and left eye pupil constriction from consensual stimulus and constriction latency stimulus difference between direct and consensual stimulus (Table 2).

**TABLE 1:** Study 2 pupillometry results.

	Left eye (Direct) stimulus (mean ± 2SD)	Right eye (Consensual) stimulus (mean ± 2SD)	Average of direct and consensual stimulus (mean ± 2SD)
Left eye pupil constriction (mm)	1.27 ± 0.47	1.20 ± 0.55	1.24 ± 0.50
Left eye pupil constriction latency (m/s)	285.93 ± 48.97	282.92 ± 38.55	284.42 ± 40.27

*No significant differences occurred for pupil constriction or pupil constriction latency whether direct, consensual, or average of direct and consensual stimulus.*

**TABLE 2:** Study 2 correlations for digit ratios with pupillometry data.

	Left pupil constriction (direct stimulus)	Left pupil constriction latency (direct stimulus)	Left pupil constriction (consensual stimulus)	Left pupil constriction latency (consensual stimulus)	Average of left pupil constriction from left (direct) and right (consensual) stimulus	Average of left pupil constriction latency from left (direct) and right (consensual) stimulus	Left pupil constriction difference for left (direct) versus right (consensual) stimulus	Left pupil constriction latency difference for left (direct) versus right (consensual) stimulus
Right 2D:4D	–0.13	–0.26	−0.50*	0.16	–0.31	–0.08	0.67**	−0.50*
Left 2D:4D	–0.17	–0.37	–0.36	–0.27	–0.34	–0.29	0.08	–0.24
Right 2D:4D minus Left 2D:4D	0.16	0.22	0.12	0.37	0.20	0.28	0.32	–0.06

Correlations (0.30 = weak, 0.50 = moderate, 0.70 = strong, 1.00 = perfect; negative value = inverse relationship). 2D:4D = second/index finger length (mm) divided by fourth/ring finger length; Right = right hand; and Left = left hand.*Significant (p < 0.05).**Significant (p < 0.01).

### Study 3

There was a statistically significant difference between groups for mean left 2D:4D [*F*(2,59 = 6.026), *p* = 0.004], and there was a significant difference for right 2D:4D minus left 2D:4D between groups [*F*(2,59 = 5.564, *p* = 0.006]. Bonferroni *post hoc* test revealed that mean left 2D:4D was greater for the political journalists (1.00 ± 0.04) compared to the rugby players (0.96 ± 0.04) and the orthopedic surgeons (0.96 ± 0.03). Similarly, the magnitude of difference for mean right 2D:4D minus left 2D:4D was greater for political journalists (0.04 ± 0.03) compared to the rugby players (-0.01 ± 0.04) and the orthopedic surgeons (0.01 ± 0.04; Table 3).

**TABLE 3:** Study 3 digit ratio comparisons between groups.

	Rugby players (*n* = 39; mean ± 2SD)	Orthopedic surgeons (*n* = 16; mean ± 2SD)	Political journalists (*n* = 18; mean ± 2SD)
Right 2D:4D	0.96 ± 0.03	0.97 ± 0.04	0.96 ± 0.04
Left 2D:4D	0.96 ± 0.04*	0.96 ± 0.03*	1.00 ± 0.04
Right 2D:4D minus Left 2D:4D Difference	–0.01 ± 0.04*	0.01 ± 0.04*	–0.04 ± 0.03

*Significantly different to political journalists (p < 0.05).

## Discussion

The purpose of this research was to explore the relationship between digit ratios and high achievement via a series of small research studies. In study one we did not observe a relationship between hormone responsiveness and digit ratios. In previous studies, under high levels of stress, this was seen (Manning et al., 2014). We deliberately chose a low intensity stressor, as indicated by an overall very low cortisol response, and at this level of stress our data suggests 2D:4D is unrelated to testosterone and cortisol response. The athletes were also highly familiar with the stress, so it posed no novelty. We suggest the 2D:4D linkage may only be observed when the stress has sufficient intensity or novelty which would be better in terms of overall conservation of response, grading the response relative to the stress intensity. However, an interesting finding was the relationships between pupillometry and digit ratios, in particular the moderate to strong positive correlation seen for right 2D:4D with left eye pupil constriction difference between direct and consensual stimulus. In previous work we argue pupillometry relates to ability to receive and process information and positively related to decline in T:C (Serpell et al., 2020b), hence declining T:C predicted poorer focus and attention. Thus, data from previous research combined with data from the present study suggest that right 2D:4D may be a marker of testosterone responsivity and therefore ability to receive and process information.

No difference in right 2D:4D was seen between print media political journalists, orthopedic surgeons, and professional rugby players. However, a novel outcome was the difference between the journalists and surgeons and rugby players for right minus left digit ratio difference, with journalists’ difference to both orthopedic surgeons and professional rugby players being significant. Previous work has linked this right and left hand 2D:4D difference to reactive aggression and masculinity (i.e., previous work has linked 2D:4D to being more “male like”; Coyne et al., 2007; Kilduff et al., 2013a). Aggression is considered a trait behavior (Serpell et al., 2020a). Journalism is a highly competitive field and often involves physical and psychological jostling for priority to stories, however, it would be hard to compare the so-called media scrum to an actual professional rugby scrum. Nevertheless, it should be considered that differences in competitive behavior of these two populations could be related to the competitive education and social role of these two figures. However, it may also be possible that successful journalism can be driven by aggression in non-physical behaviors. Australian journalists are high on extraversion and average for neuroticism (Henningham, 1997), linking to impulsive like behaviors (Perez Fuentes et al., 2016), and aggression (Bartlett and Anderson, 2012; Perez Fuentes et al., 2016).

Our results suggest that digit ratios may be indicative of androgen system responsiveness and associated behavior. Given testosterone may increase focus (Serpell et al., 2019, 2020b; Serpell and Cook, 2021), it could be argued that digit ratio is predictive of an ability to focus, a skill that would predispose to achievement in broad areas. This theory is somewhat consistent with work which has linked 2D:4D with academic performance (Coco et al., 2011; Tektas et al., 2019), and also consistent with work from our own lab which has shown that performance may be linked to testosterone via behavioral mechanisms (Cook et al., 2021). That is, digit ratio could be linked firstly to traits, and more secondary to states. These results also align more generally with other work which shows humans are a primate and performance and many behaviors can be driven, or explained, by biological or evolutionary processes (Claessens et al., 2020; Burch and Widman, 2021).

It is important that results from this study are also considered in the context of its limitations. Firstly, sample size was relatively small. However, this was an exploratory study only which was designed to help form a directional hypothesis for future work. It is worth noting, however, that other 2D:4D studies sample size range from similar to ours to hundreds, and we believe our work simply supports and grows these larger population studies. Few studies have combined digit ratio with other scientific measures (e.g., a training study measuring hormone responsiveness and pupillometry), and few have sampled professional and unique populations such as this study (professional rugby players, trained consultant orthopedic surgeons, and experienced print media political journalists). Furthermore, our statistical analysis was robust and enabled sound comparisons and relationships to be drawn. Nevertheless, we concede future work may benefit from recruiting larger samples. Though it should be acknowledged that it would be difficult to recruit large samples from these populations and apply scientific measures such as those used in this article. Secondly, although our methodology and measurement tools were robust and highly sensitive, we did not associate digit ratio to any other valid psychological or personality test; our argument that digit ratio could be linked firstly to traits, and more secondary to states may be strengthened had we. Future work may benefit from doing so. Finally, we did not recruit from a traditionally typically “artistic” population. To the knowledge of the researchers there is no known link between 2D:4D and creativity. It would be valuable to explore digit ratios in such populations. Creativity is broadly proposed as involving the ability to rapidly switch between diffuse modes and focused modes of thinking. Speculatively from a population evolutionary perspective it is easy to see the advantage of broad androgenic sensitivities, marked by 2D:4D, allowing a wide expression of talent, skills, and traits.

## Conclusion

Research has shown that digit ratios may be reflective of performance in a number of domains. We argued that this could be because those with low 2D:4D ratios are likely to have greater androgenic sensitivity, leading to an ability of maintaining focus on certain tasks, a general feature of many achievements. Therefore, we argued that 2D:4D is likely related to trait behaviors rather than limited to a specific activity or profession. Future work would benefit from recruiting larger samples and recruiting from a greater diversity of populations including those which require increased creativity for success. It may also we useful to associate digit ratios from different populations to some valid psychological or personality test.

## Data Availability Statement

The raw data supporting the conclusions of this article will be made available by the authors, without undue reservation.

## Ethics Statement

The studies involving human participants were reviewed and approved by University of Canberra Human Research Ethics Committee. The patients/participants provided their written informed consent to participate in this study.

## Author Contributions

BS and CC contributed equally to the design, collection and analysis of data, and manuscript preparation. Both authors contributed to the article and approved the submitted version.

## Conflict of Interest

The authors declare that the research was conducted in the absence of any commercial or financial relationships that could be construed as a potential conflict of interest.

## Publisher’s Note

All claims expressed in this article are solely those of the authors and do not necessarily represent those of their affiliated organizations, or those of the publisher, the editors and the reviewers. Any product that may be evaluated in this article, or claim that may be made by its manufacturer, is not guaranteed or endorsed by the publisher.
